# The oscillatory features of visual processing are altered in healthy aging

**DOI:** 10.3389/fpsyg.2024.1323493

**Published:** 2024-02-21

**Authors:** Mélanie Lévesque, Martin Arguin

**Affiliations:** ^1^Département de Psychologie, Centre interdisciplinaire de recherche sur le cerveau et l’apprentissage, Université de Montréal, Montréal, QC, Canada; ^2^Centre de Recherche, Institut Universitaire de Gériatrie de Montréal, Montréal, QC, Canada

**Keywords:** vision, aging, perceptual oscillations, object recognition, word recognition, classification images

## Abstract

The temporal features of visual processing were compared between young and elderly healthy participants in visual object and word recognition tasks using the technique of random temporal sampling. The target stimuli were additively combined with a white noise field and were exposed very briefly (200 ms). Target visibility oscillated randomly throughout exposure duration by manipulating the signal-to-noise ratio (SNR). Classification images (CIs) based on response accuracy were calculated to reflect processing efficiency according to the time elapsed since target onset and the power of SNR oscillations in the 5–55 Hz range. CIs differed substantially across groups whereas individuals of the same group largely shared crucial features such that a machine learning algorithm reached 100% accuracy in classifying the data patterns of individual participants into their proper group. These findings demonstrate altered perceptual oscillations in healthy aging and are consistent with previous investigations showing brain oscillation anomalies in the elderly.

## Introduction

Visual function is subject to several forms of alterations in normal aging. Many aspects of the visual system decline with age, but their susceptibility as well as their rate of decline may be quite variable (Faubert, 2002). The light intake of the eyes is reduced in the elderly due to a reduction of pupil size and increased thickness of the lens, whose spectral transmission function is also shifted in aging (Grossniklaus et al., 2013; Kiel et al., 2022). Functionally, studies have shown age-related deteriorations in the domains of contrast sensitivity (Owsley et al., 1983; Ross et al., 1985; Elliot, 1987; Tang and Zhou, 2009; Allard et al., 2013), visual acuity (La Fleur and Salthouse, 2014), visual overload (Scialfa et al., 2013), and motion perception (Faubert, 2002; Owsley, 2011; Legault and Faubert, 2012). It has been proposed that the more complex the visual processing required for a task, the greater the likelihood of detecting a deterioration with age (Faubert, 2002; Bertone et al., 2011; Legault and Faubert, 2012).

A feature of brain activity that has attracted an increasing degree of interest and that may help understand the deterioration of perceptual function in aging is brain oscillations. These reflect the synchronized firing of collections of neurons which are assumed to work together in achieving a micro-goal that is part of the processing required during the realization of a particular task (Buzsàki, 2006; Basar, 2013). These synchronized action potentials occur for a limited duration and vary in terms of their temporal frequency and region of origin depending on a variety of factors that are beyond the scope of the present review.

Previous studies have shown alterations of brain oscillations in aging which can be related to particular aspects of the processing required to carry out various perceptual, cognitive, or motor functions (e.g., Sebastian and Ballestros, 2012; Wiegand et al., 2013; Dushanova and Christov, 2014; Vlahou et al., 2014). Such alterations appear pervasive and can be demonstrated in a vast variety of task contexts (Babiloni et al., 2009; Sebastian and Ballestros, 2012; Dushanova and Christov, 2014; Vlahou et al., 2014; Borghini et al., 2018; Tafuro et al., 2019; Jafari et al., 2020).

In the present study, we aim to examine the behavioral manifestations of altered neural oscillatory mechanisms in aging in the context of a visual task. Indeed, an interesting implication of neural oscillations as a major basis for the functional output of our brain activity is that this output should somehow be modulated through time. With specific reference to visual function for instance, this would imply some temporal heterogeneity of our visual processing capacity. There exists a relatively substantial literature on this issue which has more or less successfully attempted to demonstrate that visual function oscillates through time at a particular unique frequency or combination thereof, which would be tied to the underlying brain activity (see Keitel et al., 2022; for a recent review).

Recently, our laboratory has developed a promising technique called random temporal sampling which offers a strong demonstration of variations of visual processing efficiency through time; i.e. perceptual oscillations. In previous studies from our lab, random temporal sampling has shown it can reveal differences in the oscillatory mechanisms called upon according to task demands (Arguin et al., 2021), the spatial frequency content of stimulation (Bertrand-Pilon and Arguin, 2023), letter position in a word recognition task (Arguin and Fortier-St-Pierre, 2023), or whether adult participants suffer from ADHD (Pelland-Goulet et al., 2023). The random temporal sampling technique involves the brief (e.g., 200 ms; allowing only one ocular fixation) presentation of stimuli made of an additive combination of signal (the target stimulus) and noise (a patch of visual white noise superimposed on the signal) and a temporal sampling function made by random variations of the signal-to-noise ratio (SNR) throughout exposure duration. By separating the temporal samples associated with errors versus correct responses and by subtracting one from the other, one obtains a classification image (CI) that characterizes processing efficiency according to the particular temporal features one wishes to consider.

For instance, Arguin et al. (2021) were able to represent variations of visual processing efficiency according either to the temporal dimension alone or as a function of a time-frequency representation of the temporal samples (i.e., temporal variations in the oscillatory power of the SNR at a range of frequencies). Moreover, they showed that the power spectra of these CIs could be successfully used by a machine learning algorithm to map these patterns of temporal features onto the particular class of stimuli participants had to recognize. Specifically, the four-way mapping of individual patterns of temporal features to the task of recognizing words, familiar objects, unfamiliar objects, or faces was performed by the algorithm with an accuracy of 75%, which is far above chance.

In the present study, we contrasted young adults (18–35 y.o.) versus elderly (60–85 y.o.) normally functioning individuals in terms of the temporal features of their visual processing in the context of an object (Exp. 1) or word recognition (Exp. 2) task. To briefly summarize the outcome of the study, we report important differences between groups in terms of the temporal features characterizing their visual processing for both tasks. Moreover, individual temporal patterns within groups are highly consistent, such that a machine learning algorithm can successfully determine whether a participant is young or elderly with 100% accuracy on the basis of an extremely small proportion of the features from these temporal patterns.

## Exp. 1: object recognition

### Methods

#### Participants

Thirty two participants, divided into two groups of 16, one with young adults (21–33 years old) and one with elderly individuals (63–80 y.o.), with normal or corrected vison (20/20) and French as their mother tongue, took part in the study. All participants gave their informed consent to participate and received a $30 CDN compensation for their participation. The study was approved by the CIUSS du Centre-Sud de Montréal Research Ethics Committee on Aging and Imaging. This approval was recognized by the Comité d’Éthique de la Recherche en Éducation et Psychologie (CÉREP) of the Université de Montréal.

For the young-adults group, participants had to be free of neurological and/or psychiatric disorders. In the end, 10 women and 6 men made up this group. Their average age was 24.1 y.o., and only one participant was left-handed. For the elderly group, participants had to report normal cognitive function. This group consisted of 12 women and 4 men, with an average age of 72.8 y.o., and only one left-handed participant.

All participants were administered the cognitive screening test Cognistat (Kiernan et al., 1987; Arguin et al., 2020) in order to verify that groups were matched on their levels of cognitive function. The mean results for each group and each subtest are reported in Table 1. T-tests comparing both groups on each subtest of Cognistat only showed a significant difference on the constructional praxis subtest (t(30) = 6.2; *p* < 0.05), where the elderly participants obtained a slightly lower mean than the young adults (5.4 vs. 6.0). Although significant, this difference remains small, and the cognitive functions involved in the praxis test are quite different from those involved in visual object recognition. Indeed, the test requires participants to replicate geometric patterns using tiles. The manipulation of small objects as well as the fact that the test is timed are most likely the factors involved in this group difference. In addition, it is worth noting that on the Naming subtest (involving the naming of images of everyday objects) both groups obtained identical scores of 8.0. The difference between the groups on the Praxis subtest is therefore not expected to have any impact on the results of the experimental task.

**Table 1:** Mean and standard deviation of the scores obtained by the young and elderly participants of Exp. 1 on each subtest of Cognistat. The numbers in parenthesis beside subtest names indicate the maximum possible score.

Subtest	Young Adults		Elderly	
	*Mean*	*SD*	*Mean*	*SD*
Orientation (/12)	12.0	0.0	11.9	0.3
Attention (/8)	7.9	0.3	7.9	0.5
Lang. Understanding (/6)	6.0	0.0	5.8	0.5
Lang. Repetition (/12)	12.0	0.0	12.0	0.0
Image naming (/8)	8.0	0.0	8.0	0.0
Construction (/6)	6.0	0.0	5.4	0.9
Memory (/12)	11.6	0.9	10.9	2.5
Calculation (/4)	3.8	0.5	4.0	0.0
Similarities (/8)	7.9	0.5	7.9	0.5
Judgment (/6)	5.8	0.5	5.6	0.8

#### Materials and stimuli

For the familiar object recognition task, stimuli were greyscale photographs (from the Bank of Standardized Stimuli; BOSS) of 256 objects. These were selected on the basis of norms collected from French-speaking participants (Brodeur et al., 2010), with the aim of maximizing the ease of finding the name, as indicated by the available statistics. Two hundred and forty images were used for the five experimental blocks of 240 trials each and a set of 16 different images were used for the practice block which was run at the very beginning of the experiment.

Images were presented in grayscale on a white background in a 640 × 640 pixel frame. Within this frame, the maximum object size was 17.4 degrees of visual angle horizontally and 17.1 degrees vertically. The minimum object size was 2.6 degrees horizontally and 3.3 degrees vertically.

The experiment was run on a Lenovo ThinkStation P52030BE computer with a 120 Hz refresh rate. The screen was calibrated to allow a linear manipulation of brightness. The corrected luminance table contained 235 values ranging from 1 to 322 lux. The viewing distance was approximately 57 cm. The head position of participants was not fixed by a chin rest or any other device. This allowed participants to be more comfortable throughout the entire experiment. The experimental program was written in Matlab and made use of functions from the Psychophysics Toolbox (Kleiner et al., 2007).

#### Procedure

On each trial, participants were asked to name aloud the object presented. The experimenter then entered the response via the computer keyboard. The dependent variable measured was the accuracy of responses. Each object image was presented once in each of the five experimental blocks. Prior to the experimental blocks, a practice block of 16 trials was administered. For the experimental and practice blocks, stimuli were presented in a different random order for each participant.

Images of the stimuli were presented for 200 ms and visual white noise was superimposed on them. The contrast of the target (the signal) was adjusted to maintain the correct response rate at about 50%. The white noise patch constituted the “noise” component of the stimulus, and its contrast was maximal (i.e., made of white and black dots). At the beginning of the experiment, the contrast of the target image was 40% of its original value. Then, from the 11^th^ trial onwards, if the participant had performed below 50% in the previous 10 trials, the target contrast was increased by one step. On the contrary, if this performance was greater than 50%, the target contrast was decreased by one step. The initial step size was 16%, and it was halved each time the adjustment of noise level changed direction until its minimum value of 1 was reached. The state of the algorithm at the end of an experimental block was retained for use at the start of the next block. The white noise patches used were constant for one trial and varied randomly across trials.

**Figure 1:**
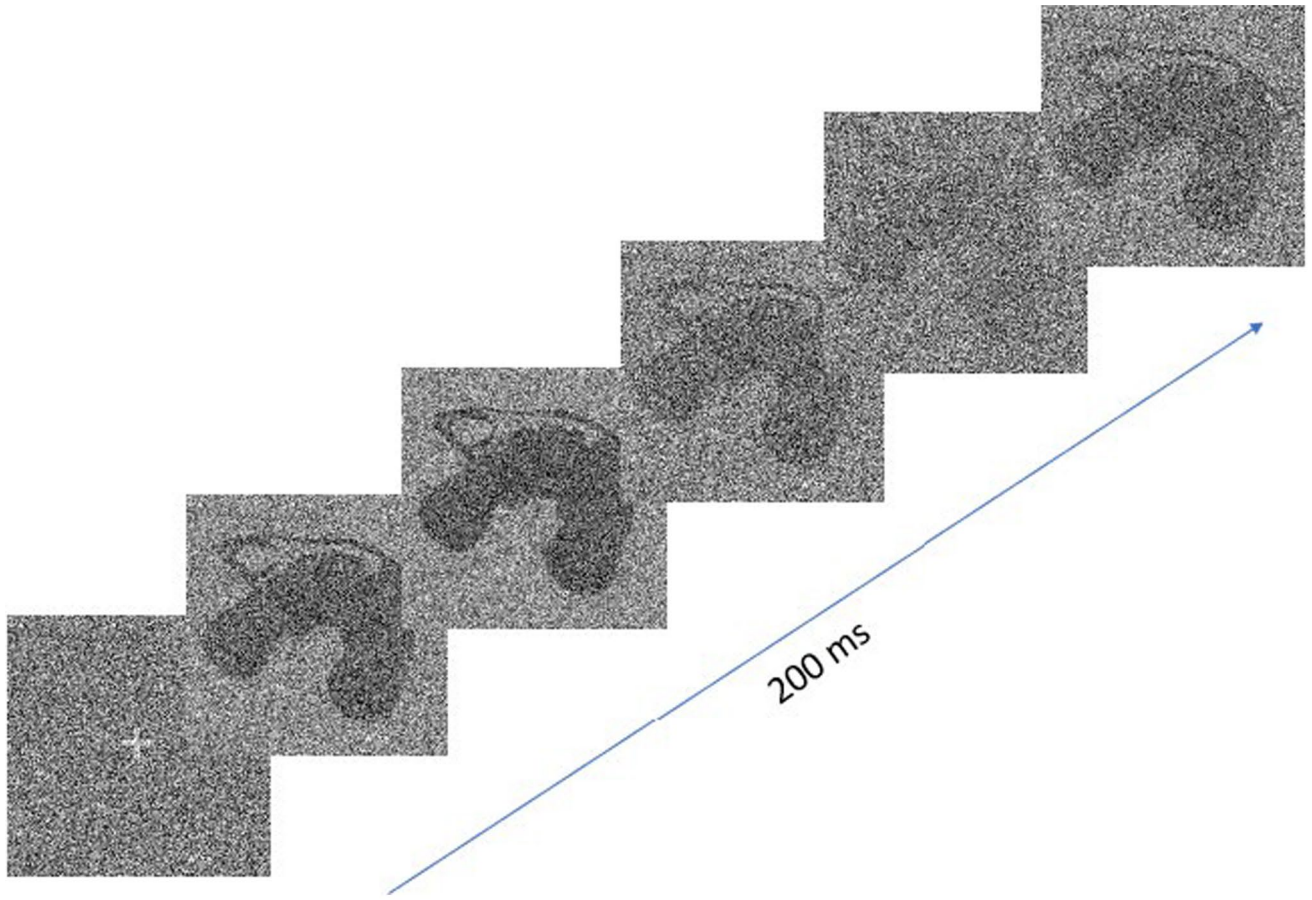
Extract from an image sequence for a trial which illustrates the change in target visibility through time.

The stimulus presented on the screen was constructed by a linear combination of the target (the signal) and random white noise. The SNR varied across the duration of exposure according to a sampling function created by integrating sine waves with frequencies between 5 and 55 Hz (in 5 Hz steps), whose amplitudes and phases were random. The SNR was normalized in the range 0 (only visual noise was visible) to 0.8 (the signal was partially obstructed by visual noise), and a new sampling function was generated for each trial. The stimulus energy was kept constant, i.e., all the images making up the sequence presented during a trial had an RMS contrast normalized to 1. Figure 1 shows an example of the change of target visibility through time which resulted from the above procedure. Given the 200 ms duration of the target and the 120 Hz temporal frequency offered by the stimulus screen, each trial consisted of a sequence of 24 images.

The experiment was conducted in two sessions, each lasting approximately one to one-and-a-half hours, which could be held on the same day or on separate days, with a maximum of one week between the two sessions. The Cognistat cognitive screening test (see above), the practice block, and two experimental blocks were administered during the first session, and the remaining three experimental blocks during the second session.

Each trial began with a 17.7 cm by 17.7 cm square patch of white noise, which was presented for 750 ms. A fixation cross was then added for 250 ms and then removed. There was a 150 ms delay before the presentation of a 900 Hz—75 dB tone for 50 ms. Following the tone, there was an additional 100 ms delay which was then followed by the onset of the target for 200 ms (i.e., the sequence of 24 images with varying SNR). Target offset was followed by the same white noise field as that presented at the beginning of the trial and it remained visible until the participant’s response. Participants could respond without time pressure, and the experimenter pressed the “1” key on the keyboard for a correct response and “0” for an error. A 1,000 Hz tone lasting 100 ms was emitted following correct responses, and a 300 Hz tone lasting 300 ms was emitted following an error.

#### Data analysis

Classification images (CIs) representing how response accuracy was affected by the temporal features of the sampling functions of the stimuli were constructed. Here we focus on CIs made from time-frequency representations of the SNR sampling functions. These were calculated separately for each trial with a wavelet analysis using three-cycle complex Morlet wavelets varying in temporal frequency from 5 to 55 Hz in 5 Hz steps (Cohen, 2014). The number of cycles in the Morlet wavelet serving as the kernel was chosen to favor high precision on the time dimension. This leads to a sacrifice in the precision of measurements in the frequency domain, which implies a sensitivity of the wavelet not just to its own temporal frequency but to a range of frequencies around it.

CIs were obtained for each individual participant. This was done by the weighted subtraction of the sums of the time-frequency sampling functions associated with errors from those associated with correct responses. These raw CIs were then transformed into Z scores by a bootstrapping operation whereby the sampling functions were randomly assigned to response accuracies while allowing for repetition, and from which sham CIs were constructed. The mean and standard deviation of 1,000 such sham CIs for an individual participant served as a reference to transform the values from their raw CI into Z scores.

Once transformed to a common scale, the individual CIs were averaged, smoothed, and then submitted to a two-way Pixel test (Chauvin et al., 2005) with α = 0.05 to determine the points in CIs that differed significantly from zero. The Pixel test is derived from random field theory and has been applied for about the last 30 years for the analysis of brain imaging data. Its purpose is to establish the Z value that will serve as the significance criterion for a Z-scored image. The smoothing filter was Gaussian and had a full width at half maximum (FWHM) of 19.6 ms in the time domain and 11.8 Hz in the frequency domain. The criterion Z score obtained was then used in its positive value to identify points in the CIs that were significantly above 0 and in its negative value (i.e., Z_crit_ *−1) to identify points significantly below 0.

The conversion of individual CIs into their Fourier descriptors was performed by one-dimensional fast Fourier transforms applied to the Z score amplitude variations through time separately for each temporal frequency represented in the time-frequency CIs (i.e., 5–55 Hz in 5-Hz steps).

The assessment of the distinctiveness of data patterns across groups was done using linear support vector machines (SVMs; Vapnik, 1995) and a leave-one-out cross-validation procedure which were applied to either the individual z-scored time-frequency CIs or their Fourier transforms. Thus, a subset of features (see below) from all but one of the available CIs were presented to the SVM for it to learn the mapping from individual CIs to their proper group (i.e., young vs. elderly). Then, the CI that had been left out of the learning phase was presented to the SVM for it to determine the group of participants it came from. This process was repeated by leaving out a different CI on each iteration until it had iterated through the complete collection. Classification accuracy was determined from the percentage of iterations on which the SVM responded correctly. Binomial analyses were used to assess whether classification accuracy deviated significantly from chance.

The classification of data patterns using an SVM satisfied several important aims in the present context. The most obvious is that an accuracy that is greater than chance implies that there exists important (i.e., significant) differences in the data patterns that are contrasted. Less obvious but crucially important is that it also provides an indication that these data patterns are replicable across individuals. Indeed, even if the average data patterns compared are markedly different, if they are not replicable across individuals, the performance of the classifier will be poor. In other words, to obtain a highly accurate classifier, the relevant features in the training patterns must retain their discriminant value in the pattern that is used in the test phase. Finally, another interesting aspect of using a classifier is that we can determine the features in the data patterns from which its discriminatory power is derived. This thus permits us to specify the feature values that characterize the groups under study. Here, we display only the characteristic features of the elderly group since those of the young participants have the same absolute value but an opposite sign.

In order to retain only the most relevant features that discriminate among conditions, we used a stepwise procedure for the introduction of features to the model one at a time, in a way that resembles the technique of stepwise multiple regression. This iterative procedure was interrupted when the classifier obtained a performance of 100% correct or when all the available features were used, whichever occurred first.

The order in which CI features were introduced to the SVM model was based on the individual capacity of each available feature to discriminate between the groups. This discrimination capacity was analogous to an F ratio; i.e. it was measured by the ratio of the variance of the means across groups over the error variance. Thus, the feature with the greatest discrimination capacity was entered first, followed by the second greatest, and so on, until the stopping criterion was reached.

For the illustration of the characteristic features of a group of participants, the only data retained was that pertaining to the features used when the SVM reached an accuracy at or above 90% correct (instead of 100% correct to avoid overfitting). The representation of a feature at each level of a factor was based on the squared difference between its mean and the overall mean across groups, which was divided by the error variance (see above). These values were then linearly normalized in the range −1 to 1 based on the maximum absolute value among the features to illustrate. To facilitate focussing on the strongest levers for classification, i.e., the features with the most extreme values, the contrast of the color code used to illustrate feature values was linearly diminished according to their distance from the extremes of the scale (i.e., −1 or 1), down to a minimum of 20% (to maintain visibility of even the weakest features illustrated). However, when the value of a feature was exactly 0, it was omitted from the figure.

### Results

The average correct response rates were of 50.5% for the young and 50.1% for the elderly [t(31) = 1.9; *ns*]. The mean contrast of target images was 12.2% for the young group and 15.35% for the elderly group, a difference which was significant [t(30) = 3.7; *p* < 0.001]. This difference is congruent with the notion of a lower degree of visual function in the elderly.

As indicated above, each object picture used in the present experiment was presented five times to each participant, thus allowing the possibility of a repetition priming effect. To assess this and determine whether groups of participants differ in this regard, a mixed factor ANOVA with groups and target repetition as factors was conducted on the response accuracy and target contrast data (Table 2). The results show a significant effect of repetition (i.e. priming) on both dependent measures (accuracy: F(4, 120) = 4.9; *p* < 0.005; contrast: F(4, 120) = 130.3; *p* < 0.001) but no interaction of repetition with group (F(4, 120) < 1; for both accuracy and contrast). These results are in agreement with the literature, showing the full preservation of repetition priming effects in aging (Fleishman, 2007).

**Table 2:** Mean percent response accuracy and image contrast (in parenthesis) as a function of group and stimulus repetition in Exp. 1.

Stimulus presentation					
Group	1	2	3	4	5
Young	51.4	50.3	50.1	50.4	50.3
	(17.0)	(12.1)	(11.8)	(10.5)	(9.7)
Elderly	50.1	50.5	49.9	50.0	49.3
	(20.0)	(15.6)	(14.9)	(13.3)	(13.0)

**Figure 2:**
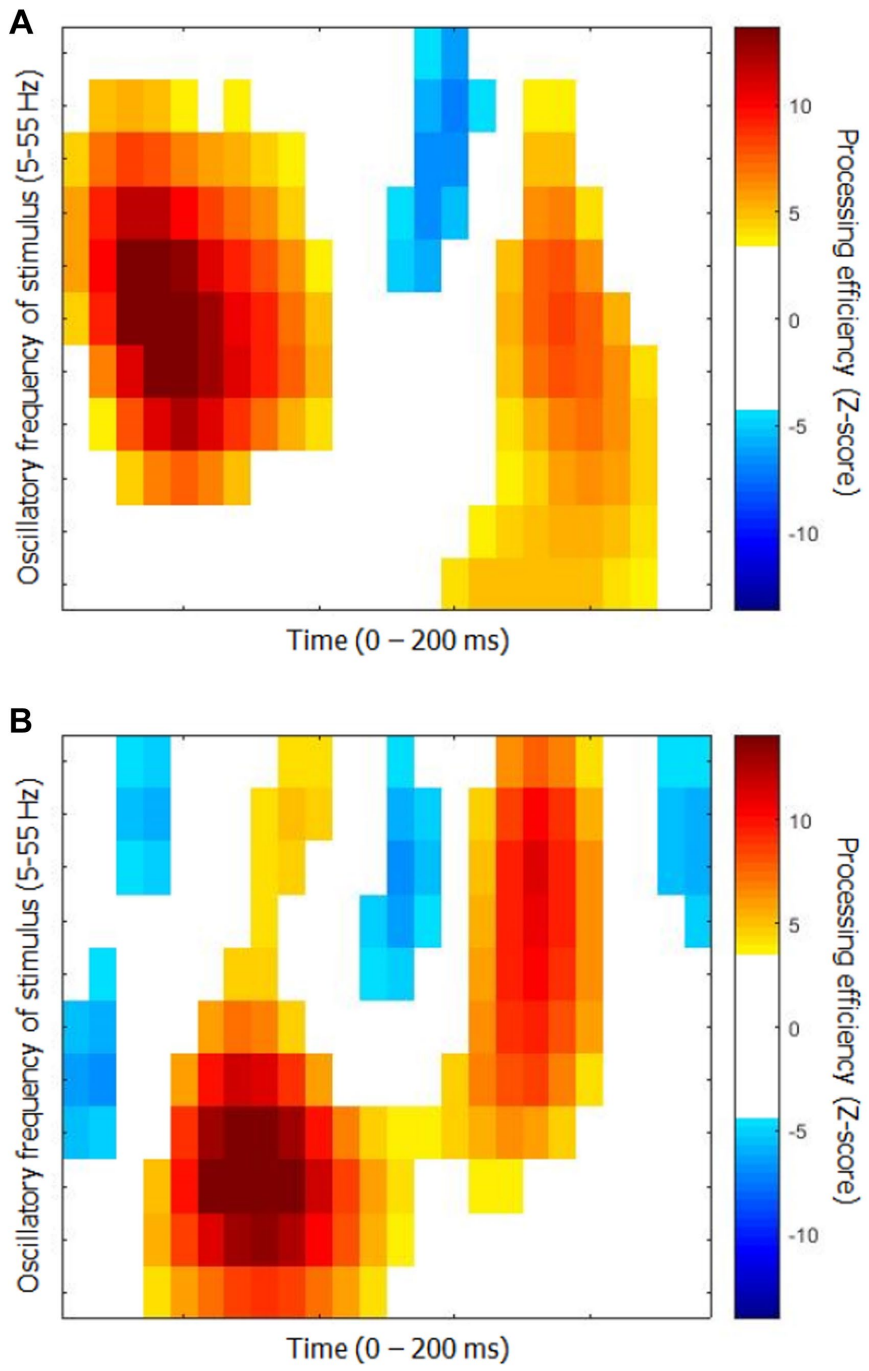
Classification images of processing efficiency as a function of time and oscillation frequencies for each group for Exp. 1 (object recognition). **(A)** Young participants. **(B)** Elderly participants. Reference for the color code is to the right of each graph. Only the points that differed significantly from 0 are in color, the others are left white.

The time-frequency CIs for each group are shown in Figure 2. They represent the capacity of participants to use the available stimulus information (i.e., processing efficiency) according to time since target onset (horizontal axis) and the oscillatory power of SNR in the frequency range 5 to 55 Hz in 5 Hz steps. While they show relatively obvious similarities in terms of the main colored blobs they contain, the positioning of these blobs within the time-frequency space is quite different. Moreover, the CI for the elderly group contains three blobs reflecting negative processing efficiencies at the very beginning and very end of the stimulation which are absent for the young participants.

An additional analysis was carried out to assess the degree to which the CIs from the two groups differ as well as to determine whether their features are shared among the members of each group. The CIs of individual participants were submitted to an SVM machine learning algorithm (with leave-one-out cross-validation), which had to determine whether the participant came from the young or elderly group. The classifier reached a maximum performance of 87.5% correct while using 13 features out of the 264 (i.e., 4.9%) making up the time-frequency CIs. This level of accuracy is well above chance (binomial test; *p* < 0.001).

**Figure 3:**
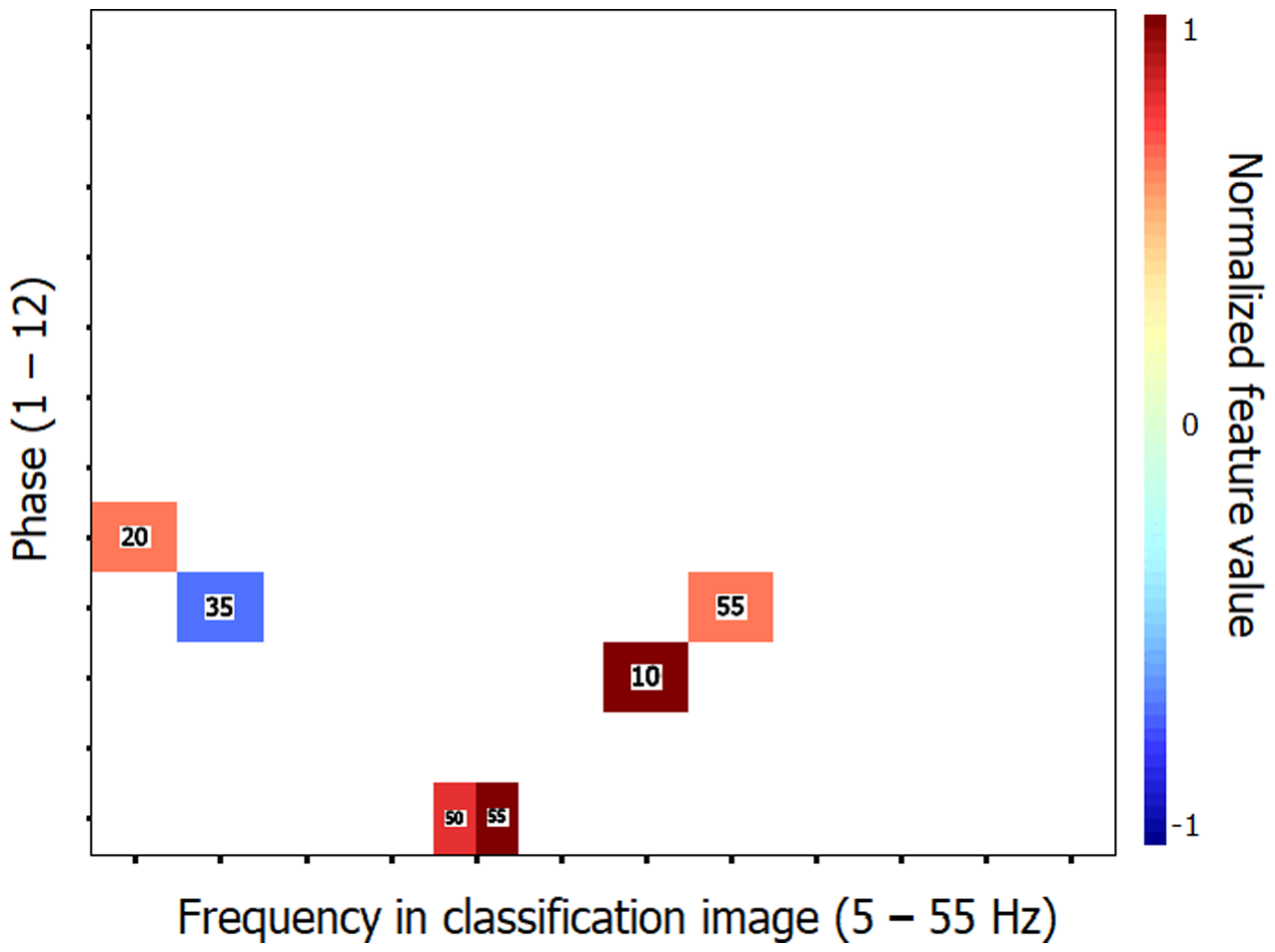
Characteristic features that distinguish the classification images (CIs) of the elderly participants from those of the young for Exp. 1 (object recognition). These features are those which supported the 90.6% accuracy of the SVM classifier in categorizing the Fourier transforms of the time-frequency CIs of individual participants according to their respective group. The horizontal axis corresponds to the temporal frequencies extracted from the CIs, the vertical axis reflects the phase values of the extracted components and the color code serves to illustrate the corresponding power values which have been normalized in the range −1 to 1 (see description of methods for details). The numbers in the colored patches indicate the frequency of target visibility oscillations that produced the Fourier features illustrated. The reader may zoom in to make them more readable. Phase × frequency cells may be occupied by more than one color patch in cases where the Fourier analysis of the CI produced two or more features contributing to the SVM with the same phase × frequency combination but which came from different stimulation oscillation frequencies.

Previous investigations in our lab using the technique of random temporal sampling and similar data analysis methods have found that classification performances were substantially better with time-frequency CIs that had been recoded into their Fourier transforms. This rule is verified again here. Thus, the SVM classifier reached an accuracy of 90.6% (binomial test; *p* < 0.001) while using only 6 of the 1,584 features (i.e., 0.4%) making up the Fourier transforms of the time-frequency CIs. The characteristic features of the elderly group that supported this level of accuracy are illustrated in Figure 3. The maximum performance attained by the classifier was 100% correct when using 32 features.

## Exp. 2: word recognition

### Methods

#### Participants

The selection criteria were identical to Exp. 1 except for the group composition. The young adult group was made of 9 women, 6 men, and 1 non-binary person. Their average age was 24.8 y.o. (range 21–33), and all of them were right-handed. The elderly group consisted of 11 women and 5 men, with an average age of 71.4 years (range 61–85), and only two left-handed participants. None of the elderly participants had taken part in Exp. 1 whereas three of the young participants had.

There was no significant difference between the groups in their performance on the various subtests of the cognitive screening test Cognistat. The mean results for each group are reported in Table 3.

**Table 3:** Mean and standard deviation of the scores obtained by the young and elderly participants of Exp. 2 on each subtest of Cognistat. The numbers in parenthesis beside subtest names indicate the maximum possible score.

Subtest	Young Adults		Elderly	
	*Mean*	*SD*	*Mean*	*SD*
Orientation (/12)	12.0	0.0	12.0	0.0
Attention (/8)	7.8	1.0	7.8	0.6
Lang. Understanding (/6)	6.0	0.0	5.9	0.5
Lang. Repetition (/12)	12.0	0.0	12.0	0.0
Image naming (/8)	8.0	0.0	8.0	0.0
Construction (/6)	5.9	0.5	5.2	1.0
Memory (/12)	11.7	0.6	10.9	2.0
Calculation (/4)	3.8	0.4	4.0	0.0
Similarities (/8)	8.0	0.0	8.0	0.0
Judgment (/6)	6.0	0.0	6.0	0.0

#### Materials and stimuli

The stimuli were 624 French five-letter words written in Courrier New with an x-size of 0.61 degrees. Six hundred words served twice each for the experimental trials (6 blocks of 200 trials each; no word was repeated within a block). A set of 24 different words were used for the practice block. The words were printed in black over a white background (prior to contrast manipulations) and their images occupied a surface of 350 × 350 pixels (9.7 × 9.7 degrees of visual angle).

#### Procedure

The procedure was identical to that of Exp. 1 except that participants were asked to name aloud the word presented on every trial.

#### Data analysis

The procedures were identical to those of Exp. 1.

### Results

The group of young participants required a contrast of the target image of 24.1% to reach an accuracy of 50.1% whereas the corresponding values for the elderly group were 31.8% contrast for 50.0% correct responses. The contrast level was significantly higher in the elderly than in the young group [t(30) = 3.9; *p* < 0.001] whereas accuracy [t(30) < 1] did not differ between groups.

To assess a possible repetition priming effect across the two presentations of each word from the list available, a mixed factor ANOVA with groups and target repetition as factors was conducted on the response accuracy and target contrast data (Table 4). The results show no significant effect of repetition or group x repetition interaction (F(1, 30) < 1; for all effects) on either dependent variable.

**Table 4:** Mean percent response accuracy and image contrast (in parenthesis) as a function of group and stimulus repetition in Exp. 2.

Stimulus presentation		
Group	1	2
Young	50.1	50.1
	(24.7)	(23.5)
Elderly	50.0	50.0
	(32.7)	(31.0)

**Figure 4:**
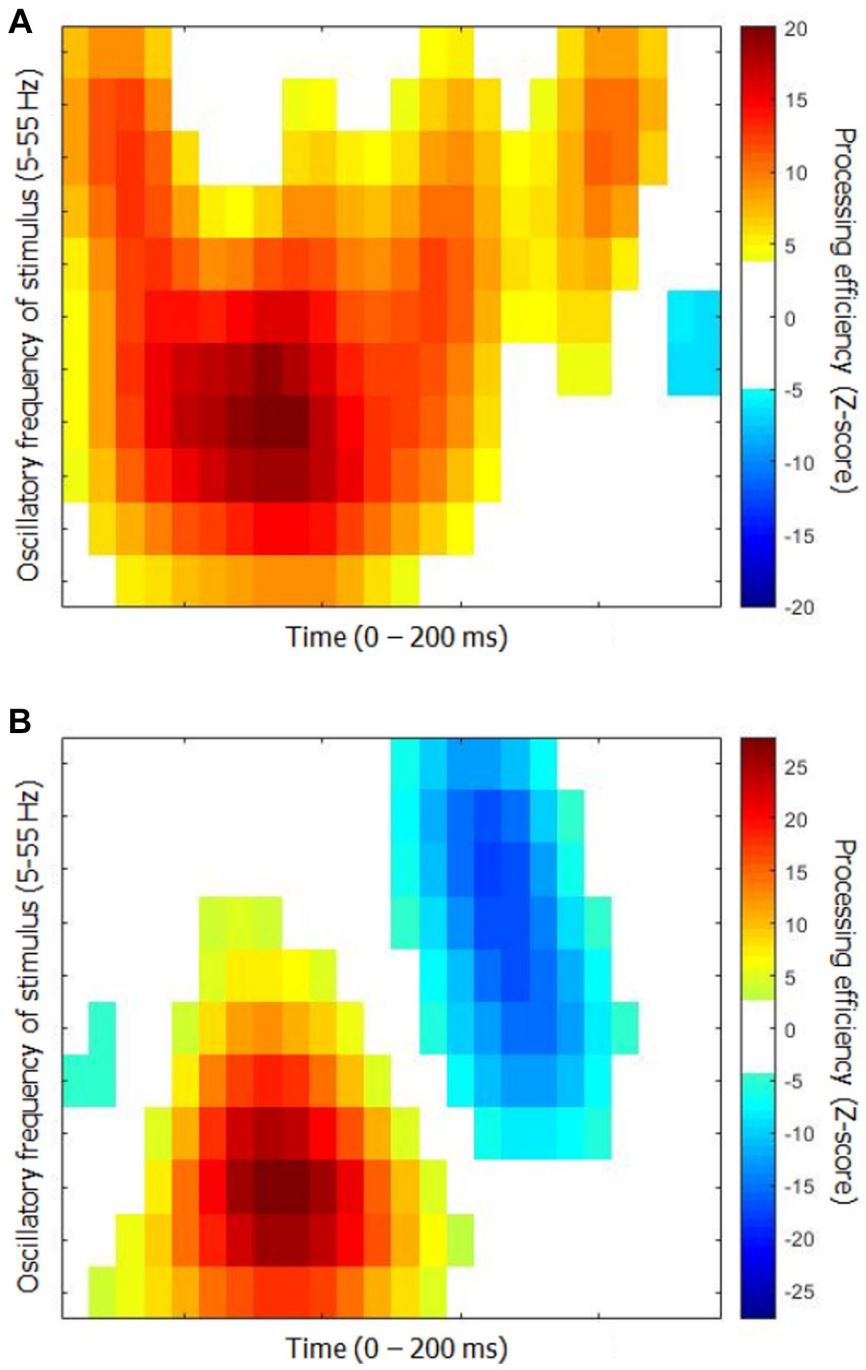
Classification images of processing efficiency as a function of time and oscillation frequencies for each group for Exp. 2 (word recognition). **(A)** Young participants. **(B)** Elderly participants. The conventions are the same as in Figure 2.

Figure 4 shows the time-frequency CIs for each group. The differences between them are quite obvious. Thus, the area corresponding to significantly positive processing efficiency is much greater for the young than the elderly group. Moreover, the latter exhibits a large segment reflecting significantly negative processing efficiency at about 140 ms after target onset which is not apparent for the young participants.

**Figure 5:**
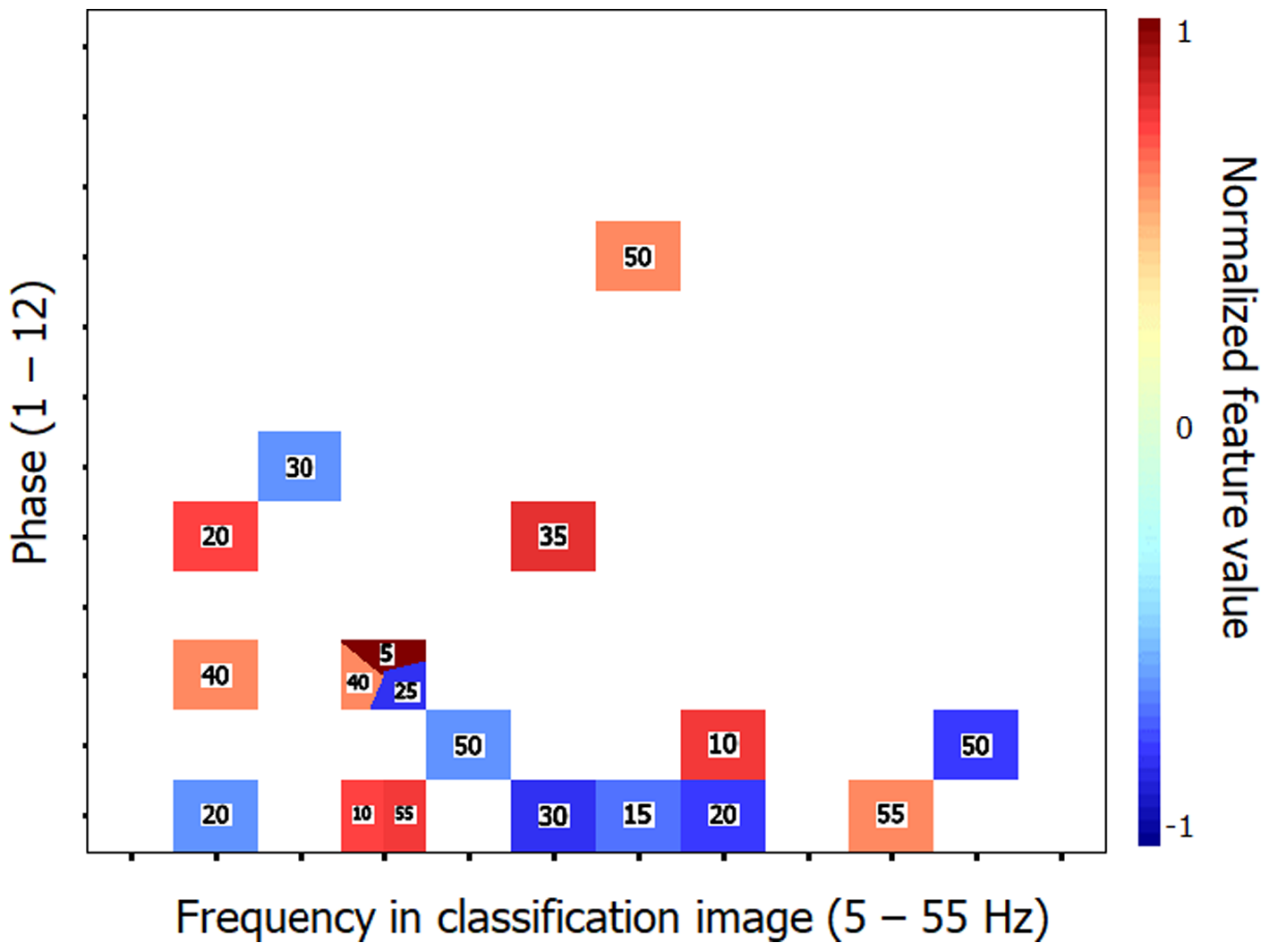
Characteristic features that distinguish the classification images (CIs) of the elderly participants from those of the young for Exp. 2 (word recognition). These features are those which supported the 93.8% accuracy of the SVM classifier in categorizing the Fourier transforms of the time-frequency CIs of individual participants according to their respective group. The conventions are the same as in Figure 3.

Similarly to Exp. 1, an SVM classifier (leave-one-out cross-validation) was exposed to the individual time-frequency CIs with the task of determining whether they were produced by a young or elderly participant. The classifier reached a performance above 90% correct (90.6%; binomial test: *p* < 0.001) while using 13 (4.9%) out of the 264 features available. Its peak accuracy was 96.9% using 28 features (10.6%). The performance of the SVM classifier was improved by exposing it to the Fourier transforms of the CIs instead of the CIs themselves. Thus, a response accuracy of 93.8% (binomial test: *p* < 0.001) was reached by using only 18 features (1.1%) out of the 1,584 making up the individual data patterns. The set of characteristic features for the elderly group that permitted this classification performance is illustrated in Figure 5. A perfect classification performance of 100% correct was obtained by exposing the classifier to 31 features (2.0%).

## General discussion

The present study examined perceptual oscillations in an object recognition task (Exp. 1) as well as in a visual word recognition task (Exp. 2) in healthy groups of young (18–35 y.o.) versus elderly (60–85 y.o.) participants using the technique of random temporal sampling. In both experiments, the level of performance was similar in the two groups, with accuracy levels very close to 50% correct. However, both for object and word recognition, the elderly group required a significantly higher level of target contrast than the young participants to reach these performance levels. This indicates that the elderly participants were somewhat impaired relative to the younger ones in carrying out these difficult tasks.

The two groups obtained CIs showing substantial variations in processing efficiency as a joint function of the time elapsed since target onset as well as the oscillatory power of SNR variations in the range of 5–55 Hz. This replicates previous observations from our lab using random temporal sampling, which showed that not only the passage of time during target exposure but also the frequency content of SNR oscillations have a large impact on processing efficiency.

While our experiments did not involve the recording of brain activity, it should be obvious that the mediation between the stimuli displayed and the responses obtained on every trial is the visual system of the participants. Thus, the classification images should be a reflection of the properties of this mediator. Since the classification images pertain to the temporal features of processing, an account thereof should rest on elements of the visual system which exhibit some form of temporal inhomogeneity in the timescale of 200 ms. Given current knowledge, the only viable candidate that we can find to account for this is that of neural oscillations. Thus, as indicated in the Introduction, we believe that the brain-activity contribution to CIs such as observed here specifically relates to the neural oscillations that generally seem to underlie functionally significant brain activity.

In Exp. 1, the differences between the CIs of each group were relatively moderate. Thus, the major elements they comprised were rather similar but they differed slightly from each other in the time of their occurrence and more substantially in the range of SNR frequencies that are involved. Despite these moderate differences, an SVM classifier had no difficulty in separating the CIs originating from either group, with a success rate of 87.5% using 4.9% of the 264 features making up these CIs. Strikingly however, the maximum success rate of the SVM classifier operating on the Fourier transforms of the CIs was 100% while using a smaller proportion (2.0%, i.e., 32 out of 1,584) of the features making up these data patterns.

In Exp. 2, the CIs of each group differed more substantially than what was found in Exp. 1. The SVM classifier was again highly successful in separating the data patterns coming from either group, with a maximum success rate of 90.6% (with 4.9% of the features available) on the individual time-frequency CIs, and a maximum success rate of 100% (with 2.0% of the features available) on their Fourier transforms.

The high level of success of the SVM classifier for both Exps. 1 and 2 has two important implications. One, more obvious, is that the CIs of the young and elderly groups show sufficiently large differences that they can be reliably discriminated on that basis. Two, less obvious but just as important, the CI features that discriminate between the young and elderly groups are largely shared among the participants of each group. Indeed, the leave-one-out cross-validation method that was used here along with the SVM classifier implies that the mapping of CI features to the groups was learned for all participants but one. The data from this remaining participant then served to test the classifier and this procedure was cycled through all individual participants. The data from the left-out participant on any cycle had to replicate the distinctive pattern that the SVM had associated with their group for the classifier to be successful. The implication then is that the distinctive features of the elderly group that were identified in each experiment (i.e., Figures 3, 5) are very stable across the elderly individuals studied here and thus, presumably, highly generalizable to other persons of the same age. On the assumption that these perceptual oscillations are a function of the neural oscillations emerging from the brain activity involved in carrying out the tasks, we should then expect that it should be possible to identify highly replicable features in the latter that would characterize healthy aging.

Regarding the performance of the SVM classifier to successfully discriminate the data patterns from the young and elderly groups, we have noted above that classification accuracy was substantially improved (i.e., greater accuracy with a smaller proportion of the features making up the data patterns available) when the CIs of individual participants were recoded into their Fourier transforms. This observation replicates a phenomenon we have observed in every single study carried out so far in our lab which jointly used the technique of random temporal sampling along with SVM classification of the data patterns (Arguin et al., 2021; Arguin and Fortier-St-Pierre, 2023; Bertrand-Pilon and Arguin, 2023; Pelland-Goulet et al., 2023). This may appear surprising considering that the information content of CIs and of their Fourier transforms is identical and that either can be recoded into the other without information loss. The account we propose for this finding is that the performance gain after applying the Fourier transform to individual CIs results from an improved compatibility of the code under which the data is represented and the temporal features of the brain activity that are responsible for performing the task. In particular, we note that recoding time-frequency CIs into their Fourier transforms leads to the elimination of the time dimension in the representation of the data, which is replaced by a phase x amplitude representation along a range of temporal frequencies (5–55 Hz). This modification leads to greatly improved similarity in the data patterns across participants who share personal features (such as age) or who perform the same task (Arguin et al., 2021).

An important additional issue that needs to be addressed concerns the possibility that the optical alterations of the eye that are associated with aging, in particular the reduced amount of light reaching the retina due to the reduction of pupil size and increased thickness of the lens, may have been a factor in the present findings. One obvious feature of such ocular changes is that they are entirely stable throughout the timescale that is relevant for the CIs reported here. As noted above, to account for the temporal features of these CIs, one needs to appeal to a property of the visual system that is temporally variable, or unstable. Since this certainly is not a feature of the reduced light intake of the eye in the elderly, we can confidently reject the possibility that this is the direct cause of the CI differences between groups. However, it remains possible that the age-related altered temporal features of the perceptual oscillations reported here may be partially attributable to perturbed neural oscillations which are secondary to the reduced light level stimulating the retina. Further studies will be necessary to directly assess this possibility.

Brain oscillations, and by extension perceptual oscillations, seem to rest in large part on the synaptic mechanisms through which neurons communicate with one another (e.g., Varela et al., 2001; Buzsàki, 2006; Plankar et al., 2012). Congruently with Vlahou et al. (2014), this leads to the suggestion that the present findings might point to altered synaptic function in healthy aging. This could be the basis for the apparent impact of processing complexity on the likelihood of observing visual deficits in normal aging (see Introduction). Specifically, the notion of processing complexity can be conceived as a proxy for the need for greater numbers of neuronal interactions in carrying out a visual task. Thus, the more synaptic events involved in a system with impaired synaptic mechanisms, the greater the risk of a processing error along the chain.

## Conclusion

The behavioral manifestations of brain oscillations, i.e., perceptual oscillations within the context of a visual task, were compared between groups of healthy young versus elderly participants. In both object and word recognition tasks, the data patterns differed substantially across groups whereas they were highly consistent within groups, such that a machine learning algorithm reached as high as 100% accuracy in classifying the results of individual participants as coming from the young or elderly group. These findings are consistent with previous demonstrations of pervasive brain oscillation anomalies in aging and they are interpreted as suggesting that healthy aging might be accompanied by altered synaptic function.

## Data availability statement

The raw data supporting the conclusions of this article will be made available by the authors, without undue reservation.

## Ethics statement

The study was approved by the CIUSS du Centre-Sud de Montréal Research Ethics Committee on Aging and Imaging. This approval was recognized by the Comité d’Éthique de la Recherche en Éducation et Psychologie (CÉREP) of the Université de Montréal. The studies were conducted in accordance with the local legislation and institutional requirements. The participants provided their written informed consent to participate in this study.

## Author contributions

ML: Data curation, Formal analysis, Investigation, Project administration, Writing – original draft. MA: Conceptualization, Data curation, Formal analysis, Funding acquisition, Methodology, Project administration, Resources, Software, Supervision, Writing – original draft, Writing – review & editing.
